# Are there employment and income gains of a national breast cancer screening program?

**DOI:** 10.1186/s13561-022-00380-0

**Published:** 2022-06-21

**Authors:** Zornitza Kambourova, Adriaan Kalwij

**Affiliations:** Utrecht University School of Economics, P.O. Box 80125, 3508, TC Utrecht, The Netherlands

**Keywords:** Breast Cancer, Screening Program, Mortality, Employment, Income, I10, I18, J21, J31

## Abstract

**Background:**

The Dutch national breast cancer screening program invites women aged 50–75 for screening. By detecting the disease in an early phase, the program aims to achieve lower breast cancer mortality and improve breast cancer survivors’ health. Arguably, the latter also improves the employability of diagnosed women.

**Objective:**

This study investigates the effects of the Dutch national breast cancer screening program on diagnosed women’s employment and income.

**Methods:**

The empirical analysis uses data of 229,357 women aged 40–59, of whom 10,515 were diagnosed with breast cancer at an age in the range 47–53. A regression-based difference-in-differences estimator is used to identify program effects by comparing outcomes for women diagnosed at ages 47–49 with the outcomes for those diagnosed at ages 50–53. The empirical models account for individual fixed effects, and for age and year fixed effects by using a control group of women who were not diagnosed with breast cancer.

**Results:**

Women’s employment rates declined in the six-year period after a breast cancer diagnosis with, on average, about 3 percentage points and their incomes declined with, on average, about 5% over this period. The empirical evidence, based on a comparison of outcomes for women diagnosed at ages 47–49 with the outcomes for those diagnosed at ages 50–53 when covered by the breast cancer screening program, does not support that these declines in employment and income were affected by the program. The evidence also does not support short or medium-term survival gains of the program.

**Conclusions:**

The findings of this study suggest that the Dutch national breast cancer screening program yields no discernible short or medium-term employment and income gains for women diagnosed with breast cancer.

**Supplementary Information:**

The online version contains supplementary material available at 10.1186/s13561-022-00380-0.

## Background

Breast cancer is the most common type of cancer for women and the second deadliest in developed countries [1]. In the Netherlands, the country analyzed in this study, one in eight women are diagnosed with breast cancer at some point in their lives [2]. The Dutch national breast cancer screening program invites women aged 50–75 for screening with the aim to improve chances of survival by detecting breast cancer in an early phase of the disease [2, 3]. Consistent with this policy objective, previous studies have reported a reduction of about 10–20% in breast cancer mortality in the Netherlands that was attributable to the program [4–7]. While the program cannot prevent breast cancer, it can lead to early detection of the disease which, in turn, can reduce breast cancer mortality and increase breast cancer survivors’ health [8]. On the premise that the program improves the health of diagnosed women, it can, arguably, also improve their employability. A quantification of these employability gains provides insights into the program’s benefits next to health improvements. Our study contributes to the literature by empirically investigating the employment and income gains of the Dutch national breast cancer screening program in the short or medium-term for women diagnosed with breast cancer.

Previous studies have shown the adverse effects of breast cancer on employment but have not empirically assessed the employment effects of a breast cancer screening program [9–13]. Such employment effects for breast cancer survivors depend on how the program affects their employability. Their employability can depend on the severity of the cancer and related intensity of the treatment that is, in turn, associated with health problems up to 5 years after the diagnosis [14, 15]. Therefore, as the program aims for an early diagnosis of the disease with less intensive treatment [3, 16], it can be argued that the previously reported adverse effects of breast cancer on employment in the Netherlands are smaller for breast cancer survivors that were covered by the program, than for those who were not covered. For instance, Danish women with more advanced stages of breast cancer experienced stronger adverse employment effects [17]. The latter finding, and the previous findings of breast cancer having an adverse employment effect, can be explained by the theoretical economic model developed by Grossman [18, 19], according to which individuals divide their time between work and leisure, and if their health deteriorates, they need time to restore it. As a result, they have less time available for work and leisure. The necessary time for recovery is, in turn, related to the severity of the health condition; the more severe the health condition, the longer the recovery time. Hence, Grossman’s model predicts that an earlier breast cancer diagnosis resulting from a screening program increases the employability of breast cancer survivors.

The Dutch breast cancer screening program is, by and large, in its current setup in place since 1998 and invites women aged 50–75 for screening once every 2 years [3, 8, 15, 20]. The screening is free of charge, is not mandatory, and involves a mammogram of the breasts at local screening units [2, 3]. Women receive an invitation for breast screening for the first time around their 50th birthday but, depending on the location of the (mobile) screening unit, some receive their first invitation around their 51st birthday while others might already receive it shortly before their 50th birthday. The program has a compliance rate of about 80%. Women under the age of 50 can also request mammography at a screening unit if they have an increased risk for breast cancer, e.g., if they have a family member who was diagnosed with breast cancer. Women whose screening results show symptoms of breast cancer, are referred to a hospital for further medical examination. The referral rate is about 2.4% of screened women and the reliability of the screening is about 82.8% [2]. Further, women diagnosed with breast cancer before the age of 50 are more likely to have been diagnosed when the symptoms of the disease appeared and are, therefore, more likely to have the disease in an advanced stage. Because the screening program aims at early detection [3 16], women diagnosed with breast cancer through the program aged 52 or older, are likely to have the disease in an early stage, as they most likely would then have been screened for a second time. Women aged 50 and 51, however, may present with the disease in either an early stage, or a more advanced stage when they are screened for the first time. Further, when diagnosed through the program, the tumor is often smaller and the cancer is less metastasized, which means that less intensive treatment options are available, for instance less frequent chemotherapy or radiation therapy in addition to breast surgery [3]. Less intensive treatment, and in particular less frequent chemotherapy, comes with less damaging side effects [15].

Our empirical analysis uses data of 229,357 women aged 40–59 for the period 2006–2012, of whom 10,515 were diagnosed with breast cancer at an age in the range 47–53. Next to information on breast cancer diagnosis, the data contains information on employment, individual income, and mortality. We analyze the effects of the breast screening program on individual income, in addition to the effects on employment, to assess if the program can mitigate possible adverse financial consequences of a breast cancer diagnosis. For identifying the program effects, we exploit that below the age of 50 women were not covered by the breast cancer screening program and were covered by the program from age 50 onwards. Our empirical models control for individual fixed effects to account for correlations between a breast cancer diagnosis and time-invariant unobserved factors of the outcome variables, and for age and year fixed effects by using a control group of women who were not diagnosed with breast cancer. Further, our analysis of the program effects on employment and income is on the premise that the program improves the health of diagnosed women. In support of this premise, previous studies have provided evidence on the program’s breast cancer survival gains [4–7]. We reexamine this evidence with the caveat that our data is on all-cause mortality and not on breast cancer mortality separately.

## Methods

### The data

Individual level administrative datasets covering the Dutch population for the years 2000–2012 were obtained from Statistics Netherlands. The datasets contain information on the years of birth and death (if deceased), household composition, gross individual annual income, employment, and information on medical diagnoses that required in-patient hospital care. Based on the latter information we can determine whether a woman was diagnosed with breast cancer in a particular calendar year. A woman’s age at diagnosis is her age on December 31 of the calendar year in which she was diagnosed with breast cancer. She is defined as being covered by the program if she was 50 years old or more on December 31 in the year of diagnosis. Further, mortality, for this study’s purposes, is established if a woman died during a calendar year.

Employment is defined as working for pay or profit [21]. Our data contains information on employment based on the main source of income during a calendar year and conditional on being alive on December 31 of that calendar year. In the Netherlands, when a woman is on sick leave she is registered as being employed. A sick leave period can, at most, be 2 years. This maximum of 2 years can be shorter if, e.g., a woman has a temporary labor contract that expires within 2 years after her falling ill, or if she is self-employed and has no insurance against an adverse health event [22]. When, after at most 2 years of sick leave, women entitled to full disability insurance benefits claim these, they are no longer registered as employed [22].

Finally, individual income is defined in terms of gross annual income from all income sources and is measured in 2012 euro. Hence, individual income includes income from employment and, e.g., unemployment and disability insurance benefits. Annual income is observed for women who were alive on December 31 of a calendar year. The degree of (social) insurance against income loss varies by type of employment, with most generous insurance for employees on permanent contracts and least generous insurance for the self-employed without private insurance against illness and disability. Social assistance provides a safety net and is means tested, hence some women left the labor force and had no income after an adverse health event and after having exhausted their (social) insurance benefits.

Our estimation sample consists of women aged 40–59 in the years 2006–2012, of whom 10,515 were diagnosed with breast cancer at an age in the range 47–53, and 218,842 were not diagnosed with breast cancer during those years and are referred to as the control group; in total there are 1,245,746 observations. The age range was restricted to 40–59 years, mainly because it is centered around the program’s age threshold of 50 and employment of women over 59 can be influenced by early retirement options [22]. The data for the years 2000–2005 were used to restrict our estimation sample to women who had not been diagnosed with breast cancer in the 6 years before entering the observation window 2006–2012. A breast cancer diagnosis in this window was considered a new diagnosis [2]. The reason for only considering a BC-diagnosis at ages 47–53 is that our analysis is based on a comparison of outcomes for women below and above the program’s age threshold of 50. The wider the range of ages at diagnosis, the less comparable are the women below and above the threshold in terms of employment, income, or health (conditional on control variables). Further, 3339 women died during the observation period, of whom 751 were diagnosed with breast cancer. Finally, employment and income are not observed in the year of death, and about 13% of women have no individual income.

A more detailed description of the data, the sample selection criteria, variable definitions, and sample means, are in Online Resource 1.

### Empirical Framework

A regression-based difference-in-differences estimator was used for estimating the effects of the Dutch breast cancer screening program on employment, mortality, and income [23]. The program effects are estimated by exploiting that women aged 47–49 were not covered by the program and women aged 50–53 were covered by the program. Furthermore, the models control for individual, age, and year fixed effects.

The basic model for estimating the effect of the program (*P*) for women who were diagnosed with breast cancer (*B*) on an outcome variable (*Y*) (is)

1 $${Y}_{it}={\alpha}_i+{\alpha}_a+{\alpha}_t+{\beta}_0{B}_{it}\times \left(1-{P}_{it}\right)+{\beta}_1{B}_{it}\times {P}_{it}+{\boldsymbol{X}}_{it}\boldsymbol{\beta} +{\epsilon}_{it}.$$

The index *i* denotes the woman and *t* denotes the year of observation. Women are followed from 2006 or age 40 onwards until the end of 2012, age 59, or the year of death (whichever came first). The dummy variable *B*_*it*_ is equal to 1 if woman *i* was diagnosed with breast cancer in or before year *t*, and 0 otherwise. The dummy variable *P*_*it*_ is equal to 1 if woman *i* was aged 50 or older in the year of diagnosis, hence covered by the screening program, and 0 otherwise. We controlled for two household composition variables, denoted by ***X***, namely household size and number of adults (the difference is the number of children). The error term is denoted by *ϵ*_*it*_. The individual, age, and year fixed effects enter the model additively and are denoted by *α*_*i*_, *α*_*a*_, and *α*_*t*_ where *a* is the age of women *i* at the end of year *t*. The effect of breast cancer on the outcome variable for women diagnosed before the age of 50 is denoted by *β*_0_, and this effect for women diagnosed at or after age 50 is denoted by *β*_1_. Hence, the effect of the program on the outcome variable is (*β*_1_ − *β*_0_).

The outcome variables are employment, mortality, and the (natural) logarithm of individual income. Hence, the former two outcome variables are modeled with linear probability models and the latter outcome variable with a linear regression model. The models were estimated by least squares and the standard errors were clustered at the individual level [24].

To investigate short and medium-term effects of the program, interaction effects between the program and the years since diagnosis are allowed for. This extended model is given by

2 $${Y}_{it}={\alpha}_i+{\alpha}_a+{\alpha}_t+{\boldsymbol{X}}_{it}\boldsymbol{\beta} +\sum_{k=0}^6{\beta}_0^k{B}_{it-k}\times \left(1-{P}_{it-k}\right)+\sum_{k=0}^6{\beta}_1^k{B}_{it-k}\times {P}_{it-k}+\sum_{a=47,a\ne 49,a\ne 51}^{53}{\gamma}_a{B}_{it}\times I\left({A}_i^d=a\right)+{\epsilon}_{it}.$$

The variable ${A}_i^d$ is the age at which breast cancer was diagnosed for woman *i*. Hence, program coverage is defined as ${P}_{it}=I\left({A}_i^d\ge 50\right)$. The effect of the program on the outcome variable *k* years after the diagnosis is $\left({\beta}_1^k-{\beta}_0^k\right)$. The last term in eq. (2) includes the ages at diagnosis and controls for possible age-related differences in the severity of the disease, with reference ages 49 and 51.

## Results

### Descriptive results

**Fig. 1:**
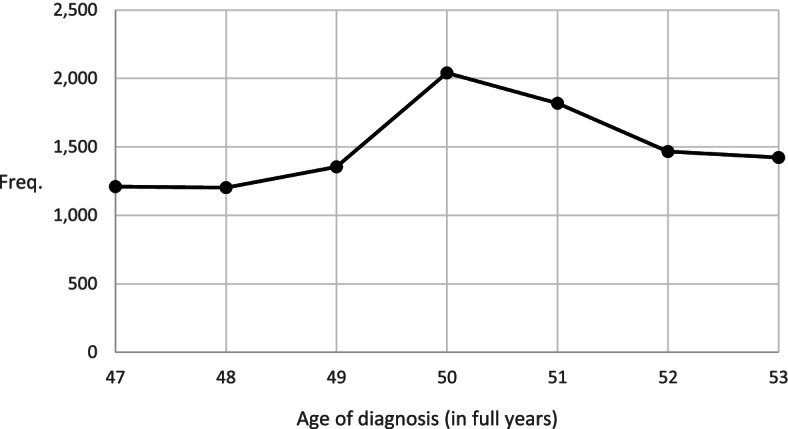
The number of women diagnosed with breast cancer by age. Notes: Age was measured on December 31 of each year and, therefore, the actual age of diagnosis was for some women 1 year lower than the age of diagnosis registered in our data. Online Resource 1 shows the numbers for this figure

Women were most likely to receive a breast cancer diagnosis at the ages of 50 and 51 than at any other age (Fig. 1). This suggests screening can detect breast cancer before the symptoms appear.

**Fig. 2:**
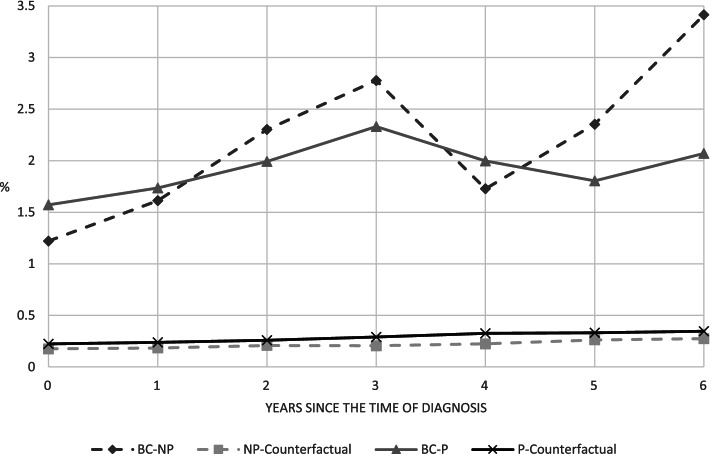
Annual mortality rates for women diagnosed with breast cancer at ages 47–53 and their counterfactual rates. Notes: The annual mortality rate is defined as the percentage of women who died during a calendar year. The four groups of women are: BC-P (BC-NP) for women diagnosed with breast cancer and who were (not) covered by the program, and P-Counterfactual (NP-Counterfactual) for women who were not diagnosed with breast cancer and in the same years were of the same ages as women in the BC-P (BC-NP) group. Online Resource 1 shows the numbers for this figure

The annual mortality rates are substantially higher for women who were diagnosed with breast cancer than for those who were not (Fig. 2). The rates for non-diagnosed women, i.e., the counterfactual rates, are in the range of 0.18–0.35%. Mortality rates for diagnosed women are in the range of 1.22–3.41%. For the latter group, the differences between women diagnosed when covered by the screening program and women diagnosed when not covered by the program are small and, tentatively, show an increase 6 years after the diagnosis.

**Fig. 3:**
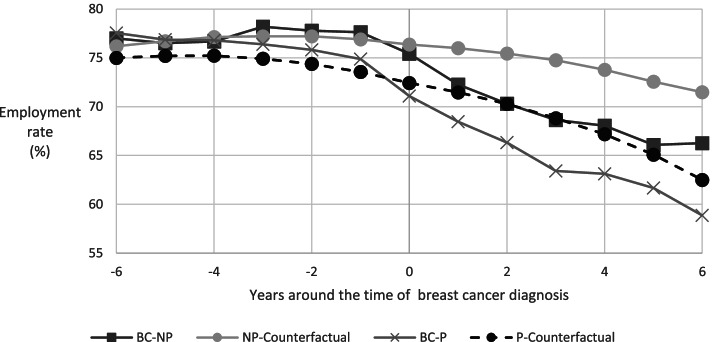
Annual employment rates for women diagnosed with breast cancer at ages 47–53 and their counterfactual employment rates. Notes: The four groups of women are: BC-P (BC-NP) for women diagnosed with breast cancer and who were (not) covered by the program, and P-Counterfactual (NP-Counterfactual) for women who were not diagnosed with breast cancer and in the same years were of the same ages as women in the BC-P (BC-NP) group. Online Resource 1 shows the numbers for this figure

From the time of breast cancer diagnosis onwards, the women’s employment rates decreased, also relatively to the counterfactual rates (Fig. 3). These decreases are rather similar for those covered and those not covered by the program. Furthermore, a comparison of the rates of diagnosed women before the time of diagnosis with their counterfactual rates show parallel trends. The declines in the counterfactual employment rates underline the importance of using a control group in our empirical analysis and to avoid overestimating the effects of a breast cancer diagnosis on employment.

**Fig. 4:**
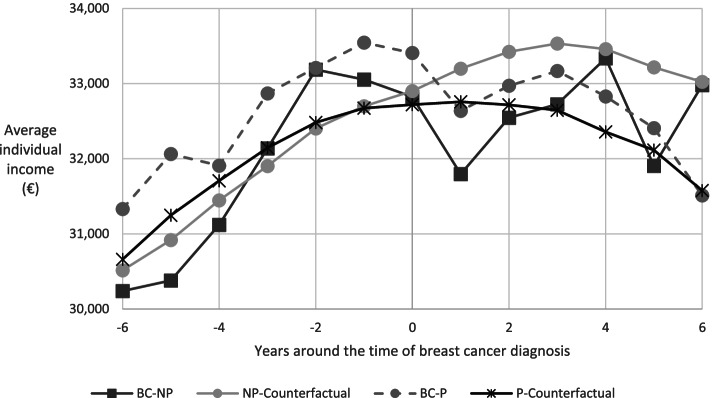
Mean individual income for women diagnosed with breast cancer at ages 47–53 and their counterfactual mean incomes. Notes: The four groups of women are: BC-P (BC-NP) for women diagnosed with breast cancer and who were (not) covered by the program, and P-Counterfactual (NP-Counterfactual) for women who were not diagnosed with breast cancer and in the same years were of the same ages as women in the BC-P (BC-NP) group. Online Resource 1 shows the numbers for this figure

Income drops in the year of breast cancer diagnosis and the following year, and recovers in the years thereafter (Fig. 4). This pattern does not seem to differ between diagnosed women who were and those who were not covered by the program. Further, there is a higher average level of income for women diagnosed when covered by the program and the average incomes before the year of diagnosis for the different groups show parallel trends. Restricting the figure to employed women yields similar patterns (not shown in a figure).

### Estimation results of eqs. (1) and (2) for employment, income, and mortality

The drop in the employment rate for women diagnosed with breast cancer when not covered by the screening program was, on average, about 3.4 percentage points (pp) (Table 1, first column, Panel A). This drop was about equal for women diagnosed when covered by the program (3.3 pp) and this is also shown by the difference in the last row of Panel A (0.001 with a standard error of 0.006). These findings are not in support of a program effect on employment for diagnosed women. Panel B, first column, shows that the drop in the employment rate gradually increased up to about 3 years after diagnosis. When allowing for differences in short and medium-term effects of a breast cancer diagnosis on employment, the evidence is not in support of program effects (first column, last rows of Panel B or Panel C).

**Table 1:** Estimation results for the program effects on employment and mortality

	Employment		Mortality	
	Coef.	Std.Err.	Coef.	Std.Err.
*Panel A: Difference from the baseline for BC diagnosed women, Eq. (**1**)*				
No screening program, *β*_0_	−0.034***	(0.005)	0.018***	(0.002)
Screening program, *β*_1_	−0.033***	(0.003)	0.017***	(0.001)
Program effect, Eq. (1): (*β*_1_ − *β*_0_)	0.001	(0.006)	−0.001	(0.002)
*Panel B: Differences from the baseline for BC diagnosed women by time since diagnosis, Eq. (**2**)*				
*No screening program*, ${\beta}_0^k$ s				
In year of diagnosis	−0.012	(0.008)	0.007**	(0.003)
1 year after diagnosis	−0.040***	(0.008)	0.014***	(0.003)
2 years after diagnosis	−0.054***	(0.009)	0.026***	(0.004)
3 years after diagnosis	−0.067***	(0.010)	0.036***	(0.004)
4 years after diagnosis	−0.065***	(0.011)	0.032***	(0.004)
5 years after diagnosis	−0.067***	(0.012)	0.042***	(0.005)
6 years after diagnosis	−0.058***	(0.015)	0.057***	(0.009)
*Screening program,* ${\beta}_1^k$ *s*				
In year of diagnosis	−0.019***	(0.007)	0.010***	(0.002)
1 year after diagnosis	−0.035***	(0.007)	0.016***	(0.003)
2 years after diagnosis	−0.047***	(0.008)	0.023***	(0.003)
3 years after diagnosis	−0.064***	(0.008)	0.031***	(0.003)
4 years after diagnosis	−0.054***	(0.009)	0.033***	(0.004)
5 years after diagnosis	−0.052***	(0.010)	0.035***	(0.004)
6 years after diagnosis	−0.048***	(0.012)	0.041***	(0.006)
*Program effect,* $\left({\beta}_1^k-{\beta}_0^k\right)$ *s*				
In year of diagnosis	−0.007	(0.010)	0.003	(0.004)
1 year after diagnosis	0.004	(0.011)	0.002	(0.004)
2 years after diagnosis	0.007	(0.012)	−0.002	(0.005)
3 years after diagnosis	0.003	(0.013)	−0.005	(0.006)
4 years after diagnosis	0.011	(0.014)	0.001	(0.006)
5 years after diagnosis	0.015	(0.016)	−0.007	(0.007)
6 years after diagnosis	0.010	(0.019)	−0.016	(0.010)
*Panel C: Specification tests (p-values)*				
No program effects	0.909		0.372	
No time since diagnosis effects	0.000***		0.000***	
No age at diagnosis effects	0.589		0.152	
Common trend before the time of diagnosis	0.981			
Hausman test (H_0_: random effects)	0.000***		0.000***	
R^2^ (within)	0.006		0.007	
Number of observations	1,242,852		1,245,746	
Number of years	7		7	
Number of women	229,011		229,357	

Notes. *BC* Breast cancer. The models control for year, age, household size, number of adults, and for panel C, age at diagnosis. For Panel C: No program effects, H_0_: $\left({\beta}_1^0-{\beta}_0^0\right)=..\left({\beta}_1^6-{\beta}_0^6\right)=0$; No time since diagnosis effects, H_0_: ${\beta}_0^0=..={\beta}_0^6$, ${\beta}_1^0=..={\beta}_1^6$, No age at diagnosis effects, H_0_: *γ*_47_ = *γ*_48_, *γ*_50_ = *γ*_52_ = *γ*_53_. The standard errors are clustered by individual. Levels of significance: *** p < 0.01, ** p < 0.05, * p < 0.10

The findings in the second column of Table 1 (all panels) do not support that the program increases breast cancer survival in the short or medium-term. The mortality rate for diagnosed women increased by about, on average, 1.8 pp. and 1.7 pp. for, respectively, women not covered by the program and those covered by the program (Panel A). Furthermore, the results show that mortality increased in the years after diagnosis and, tentatively, suggest that if there would survival gains, that these are in the long-term (Panel B).

The income of women diagnosed with breast cancer dropped, on average, by about 5.7% for those not covered by the program and 4.8% for those covered by the program (Table 2, first column, Panel A). Panel A shows that there is no empirical evidence in support of a program effect on income. When allowing for differences in short and medium-term effects, the findings also show no evidence in support of the program affecting income losses after being diagnosed with breast cancer (Panels B and C).

**Table 2:** Estimation results for the program effects on the logarithm of individual income

	Ages 47–53		Ages 49 and 51	
	Coef.	Std.Err.	Coef.	Std.Err.
*Panel A: Difference from the baseline for BC diagnosed women, Eq. (**1**)*				
No screening program, *β*_0_	−0.057***	(0.008)	− 0.052***	(0.012)
Screening program, *β*_1_	−0.048***	(0.006)	− 0.044***	(0.010)
Program effect, Eq. (1): (*β*_1_ − *β*_0_)	0.009	(0.010)	0.008	(0.016)
*Panel B: Differences from the baseline for BC diagnosed women by time since diagnosis, Eq. (**2**)*				
*No screening program*, ${\beta}_0^k$ s				
In year of diagnosis	− 0.027**	(0.013)	−0.028**	(0.013)
1 year after diagnosis	−0.082***	(0.014)	−0.076***	(0.016)
2 years after diagnosis	−0.068***	(0.014)	−0.068***	(0.016)
3 years after diagnosis	−0.057***	(0.015)	−0.064***	(0.021)
4 years after diagnosis	−0.050***	(0.018)	−0.056**	(0.027)
5 years after diagnosis	−0.030	(0.019)	−0.021	(0.024)
6 years after diagnosis	−0.022	(0.022)	−0.062	(0.030)
*Screening program,* ${\beta}_1^k$ *s*				
In year of diagnosis	−0.032***	(0.010)	−0.028***	(0.010)
1 year after diagnosis	−0.062***	(0.011)	−0.059***	(0.012)
2 years after diagnosis	−0.046***	(0.011)	−0.058***	(0.015)
3 years after diagnosis	−0.041***	(0.012)	−0.045***	(0.015)
4 years after diagnosis	−0.053***	(0.014)	−0.040*	(0.021)
5 years after diagnosis	−0.061***	(0.017)	−0.057**	(0.027)
6 years after diagnosis	−0.040	(0.022)	−0.060	(0.042)
*Program effect,* $\left({\beta}_1^k-{\beta}_0^k\right)$ *s*				
In year of diagnosis	−0.005	(0.016)	0.000	(0.017)
1 year after diagnosis	0.020	(0.017)	0.016	(0.020)
2 years after diagnosis	0.021	(0.018)	0.010	(0.022)
3 years after diagnosis	0.016	(0.019)	0.018	(0.025)
4 years after diagnosis	−0.003	(0.023)	0.016	(0.034)
5 years after diagnosis	−0.030	(0.025)	−0.036	(0.036)
6 years after diagnosis	−0.018	(0.031)	0.002	(0.051)
*Panel C: Specification tests (p-values)*				
No program effects	0.052*		0.668	
No time since diagnosis effects	0.000***		0.001***	
No age at diagnosis effects	0.651			
Common trend before the time of diagnosis	0.002***		0.255	
Hausman test (H_0_: random effects)	0.000***		0.000***	
R^2^ (within)	0.024		0.024	
Number of observations	1,086,034		1,042,603	
Number of years	7		7	
Number of women	206,186		199,627	

Notes. *BC* Breast cancer. The models control for year, age, household size, number of adults, and for panel C, age at diagnosis. For Panel C: No program effects, H_0_: $\left({\beta}_1^0-{\beta}_0^0\right)=..\left({\beta}_1^6-{\beta}_0^6\right)=0$; No time since diagnosis effects, H_0_: ${\beta}_0^0=..={\beta}_0^6$, ${\beta}_1^0=..={\beta}_1^6$, No age at diagnosis effects, H_0_: *γ*_47_ = *γ*_48_, *γ*_50_ = *γ*_52_ = *γ*_53_. The standard errors are clustered by individual. Levels of significance: *** p < 0.01, ** p < 0.05, * p < 0.10

### Model assumptions and specification tests

Exogeneity of being diagnosed with breast cancer is required for unbiasedness of the estimator of the program effects. The breast cancer risk factors for women, besides age, are related to genetics in about 8–10% of the cases, and to life-style factors such as level of education, age at first pregnancy, alcohol consumption, smoking, and the use of oral contraceptives [3, 25]. Our data has no information on such factors, besides household size, therefore, to identify a causal relationship we rely on the individual fixed effects *α*_*i*_ s to sufficiently control for such factors. The results of the Hausman tests in Panels C of Tables 1 and 2 confirm the importance of controlling for individual fixed effects [26].

The model compares outcomes of diagnosed women who are covered by the program (ages 50–53) with outcomes of those who are not covered by the program (ages 47–49). This requires controlling for age effects on these outcomes in the absence of a diagnosis, i.e., the counterfactual outcomes. Individual fixed effects control for level differences and to account for age (or trend) differences requires the assumption that the counterfactual trends for diagnosed women are the same as the trends for non-diagnosed women (conditional on, e.g., year and individual fixed effects). This common trend assumption is tested based on the outcomes for diagnosed women before they were diagnosed [23]. The test result in Panel C of Table 1 is in favor of the common trend assumption for employment. For income, however, the evidence is not in favor of the common trend assumption (Table 2, first column, Panel C). This result can be related to different income profiles for older and younger women over the observation period (Fig. 4). We, therefore, estimated the income model also for women aged 49 and 51 only. Arguably, these two groups of women have similar income profiles (before being diagnosed). These additional results are reported in column 2 of Table 2 and the test result in Panel C is in favor of a common trend. Also, the main conclusion based on column 1 remains: the evidence is not in favor of program effects on income for diagnosed women. Furthermore, narrowing the range of the ages at diagnosis for the employment and mortality models or restricting the samples for Table 2 to employed women did not affect the main findings (not shown in a table).

Further, the severity of the breast cancer tumor can vary with age [27, 28]. The test results in the rows corresponding to ‘No age at diagnosis effects’ in Panels C of Tables 1 and 2 show no evidence in support of the age at diagnosis, within the two groups defined by program coverage, affecting the outcomes. This can be interpreted as support for using the age-at-diagnosis ranges 47–49 and 50–53 for our empirical analysis.

Finally, because we found no evidence of survival gains of the program within our sample period, survival bias is unlikely to be an issue for our estimated program effects on employment [9].

## Discussion

The main empirical findings of our study are twofold. First, on average in the 6 years after a breast cancer diagnosis, women’s employment rates declined with about 3 pp., their mortality rates increased with almost 2 pp., and their incomes declined on average with about 5%. The reduction in employment after a breast cancer diagnosis is in line with earlier findings [9, 11]. Further, the drop in the employment rate gradually increased up to about 3 years after diagnosis, which is, arguably, related to employment protection for up to 2 years after an adverse health event (section 2 [29];). Second, we found no empirical support for short or medium-term effects of the Dutch national breast cancer screening program on the employment, mortality, or income of women who are diagnosed with breast cancer. The finding of no support for survival gains of the program is in contrast with the earlier findings for the Netherlands of a 10–20% reduction in breast cancer mortality [4–7, 30]. It is, however, in line with a recent finding for the Netherlands of no discernible program effects for all-cause mortality [7], which in turn agrees with the international evidence from randomized controlled trails [31].

To the best of our knowledge, this study is the first to examine the employment and income gains of a breast cancer screening program. We started this research on the premise of health and survival gains of the program. This premise can be questioned because of our finding of no discernible short or medium-term survival gains of the program. The absence such gains can be because, e.g., the gains are in the long-term or offsetting adverse effects of screening from over-treatment [7, 14, 32]. Our findings of no discernible employment and income gains of the program are in accordance with those for the survival gains and, arguably, suggest no discernible health gains of the program.

From a policy perspective, our findings do not necessarily imply that the breast cancer screening program is redundant. For instance, the mere presence of the program can cause women of all ages to be aware of breast cancer and to contact their general practitioner as soon as the symptoms appear. Such behavioral effects are not identified in our paper.

Future research using data on the health behavior and knowledge, as well as on economic and health outcomes, in relation to breast cancer diagnoses can provide insights into our findings. This poses an empirical challenge for the assessment of a screening program that needs to account for, e.g., behavior responses to the presence of a screening program or to having the option of screening at ages below the program’s age threshold. Further, information about women who do not show up for screening (the 20% noncompliers) can be used to assess if these women are less aware of the risks of breast cancer, would not go to their general practitioner when having symptoms of breast cancer, and could benefit from screening. Finally, information on breast cancer diagnoses in between screening moments can be used to assess the effectiveness of screening once every 2 years.

## Conclusions

Based on our empirical results we conclude that the Dutch national breast cancer screening program yields no discernible short or medium-term employment and income gains for diagnosed women. Arguably, a possible explanation for these results, and given we also find no discernible survival gains of the program in the short or medium-term, is that there are on average no discernible health gains of the Dutch national breast cancer screening program.

## Supplementary Information

**Additional file 1: Online Resource 1.** Data and sample selection [12, 21, 33–36].

## Data Availability

Access to the data is provided by Statistics Netherlands (www.cbs.nl). Access can be granted for academic research through the remote access facility of Statistics Netherlands. It is a secure data environment and data cannot be exported out of this environment. The statistical codes for the empirical analysis are stored in this environment.

## Abbreviations

A: Age
BC or B: Breast Cancer
P: Program (Dutch national breast cancer screening program)
Group BC-P: women diagnosed with breast cancer and who were covered by the program
Group BC-NP: women diagnosed with breast cancer and who were (not) covered by the program
Group P-Counterfactual: women who were not diagnosed with breast cancer and in the same years were of the same ages as women in the BC-P group
Group NP-Counterfactual: women who were not diagnosed with breast cancer and in the same years were of the same ages as women in the BC-NP group
