# Supplementary material for: Are there employment and income gains of a national breast cancer screening program?
Source: Health Econ Rev. 2022 Jun 21;12:33. doi: 10.1186/s13561-022-00380-0 (PMC9210695; doi:10.1186/s13561-022-00380-0)
Supplement: Supplementary file 1 — Additional file 1: Online Resource 1. Data and sample selection [12, 21, 33–36]. [file 13561_2022_380_MOESM1_ESM.docx]

**Online Resource 1** **Data and sample selection**

The data was provided by Statistics Netherlands. We retrieved the years of birth and death (if deceased), and household composition for all individuals residing in the Netherlands from the Population Registry [33]. The gross annual incomes and employment status of these individuals were obtained, by Statistics Netherlands, from various institutions, such as the tax authority and Employee Insurance Agency [34]. Finally, we obtained medical information on women’s main diagnosis, within a calendar year, that required in-patient hospital care from the National Medical Registration [35].

The raw data covers the period 2000-2012 and includes about 3.3 million women of the birth cohorts 1947-1972 (Fig. A1). About 4% of women were not residing in the Netherlands for one or more years during the 2000-2012 period; they were excluded from the sample, as no information was available for their years abroad. The medical data covered about 80% of the Dutch population over the sample period, as some hospitals did not supply medical information to the National Medical Registration [36]. For this reason, and following previous studies, we excluded about 20% of women from our sample based on municipality information [12]. The sample is restricted to the years 2006-2012. Medical information for the years 2000-2005 was used to address the issue of a recurrent breast cancer diagnosis. Women who had been diagnosed with breast cancer in the six years preceding their inclusion in the 2006-2012 sample were excluded (6,472 women). That is, if a woman had not received a breast cancer diagnosis for a period of at least six years, a breast cancer diagnosis was considered a new diagnosis. A further 9% of women were excluded because information on income was missing. To keep the empirical analysis feasible, we selected completely at random 10% of those women who had not been diagnosed with breast cancer. Finally, our estimation sample excludes women who were diagnosed before the age of 47 or after the age of 53 and consists of 10,515 women who were diagnosed with breast cancer at an age in the range 47-53 and a control group of 218,842 women who were not diagnosed with breast cancer during the observation period. Women who were diagnosed are also in the sample for the years before or after being diagnosed (if not deceased).

Tables A1 and A2 reports on the variables for the empirical analysis. Age is measured in full years on December 31 of each calendar year and a woman is registered as deceased if she died during a calendar year. The age at diagnosis is taken as the age on December 31 of the calendar year in which a woman was diagnosed with breast cancer, and she was defined as being covered by the program if she was 50 years or older on December 31 in the year of diagnosis. A breast cancer diagnosis included both invasive and in situ breast cancer and refers to the codes “174 Malignant neoplasm of female breast” and “233.0 Carcinoma in situ of breast” of the ninth revision of the International Classification of Diseases. Household size is the sum of the numbers of adults and children. A household member aged 18 years or older was defined as an adult and was otherwise defined as a child. Employment was defined as work for pay or profit [21] and only observed for women alive on December 31 of a calendar year. The employment status was based on a person’s largest source of income by Statistics Netherlands. Individual income is total gross annual income from all sources in 2012 euro, and the reported means in the tables exclude those who had no income.

Table A3 reports the numbers used for Figure 1 and Table A4 reports the numbers used for Figures 2-4.

**Additional References**

1. CBS. *Documentatierapport Huishoudenskenmerken van in de Gemeentelijke Basisadministratie Persoonsgegevens (GBA) ingeschreven personen (GBAHUISHOUDENSBUS).* Centrum voor Beleidsstatistiek en Microdata Services, Statistics Netherlands; 2015.
2. CBS. D*ocumentatierapport Integraal Persoonlijk Inkomen (IPI).* Centrum voor Beleidsstatistiek en Microdata Services, Statistics Netherlands; 2016.
3. CBS. *Documentatierapport Landelijke Medische Registratie* (LMR) 2012. Centrum voor Beleidsstatistiek en Microdata Services, Statistics Netherlands; 2016.
4. Van der Laan J. Quality of the Dutch Medical Registration (LMR) for the calculation of the Hospital Standardised Mortality Ratio. *Discussion paper 201308*, Statistics Netherlands; 2013.

**Fig. A1** Stepwise sample selection.

Population data: All residents of the Netherlands in the calendar years 2000-2012.

Include: women, birth cohorts 1947-1972.

N1= 3,318,845; N2= 41,030,216

Include: those who are residents in the Netherlands in all calendar years (when not deceased).

N1= 3,066,190; N2= 39,426,125

Include: those covered by the National Medical Registration

N1= 2,449,115; N2= 31,484,912

Include:

1. calendar years 2006-2012
2. women at ages 40-59.
3. non-BC-diagnosed women or first-time BC-diagnosed women.

N1= 2,442,602; N2= 13,106,815

Exclude: missing income data.

N1= 2,214,068; N2= 11,897,866

Include: 10% of non-BC diagnosed women and all BC-diagnosed women

N1= 243,063; N2= 1,324,114

Exclude: BC-diagnosed women if diagnosed before age 47 or after age 53.

N1= 229,357; N2= 1,245,746

Note: N1 = number of women, N2 = number of observations, BC = breast cancer.

**Table A1** Sample sizes and frequencies or sample means for women who were not diagnosed with breast cancer.

| Age | Number of observations | Deceased | Number of adults | Household size |  | Employment | Individual income |
| --- | --- | --- | --- | --- | --- | --- | --- |
|  | Freq. | Freq. | Mean | Mean |  | % | Mean |
| 40 | 59,771 | 43 | 1.85 | 3.55 |  | 79.59 | 32,467 |
| 41 | 60,344 | 68 | 1.85 | 3.55 |  | 79.28 | 32,537 |
| 42 | 60,841 | 61 | 1.84 | 3.54 |  | 79.03 | 32,576 |
| 43 | 61,006 | 52 | 1.84 | 3.52 |  | 78.66 | 32,661 |
| 44 | 60,967 | 68 | 1.84 | 3.50 |  | 78.35 | 32,648 |
| 45 | 61,223 | 86 | 1.84 | 3.46 |  | 78.00 | 32,809 |
| 46 | 61,358 | 76 | 1.83 | 3.41 |  | 77.55 | 32,924 |
| 47 | 61,445 | 98 | 1.83 | 3.34 |  | 77.02 | 32,863 |
| 48 | 61,119 | 109 | 1.83 | 3.25 |  | 76.29 | 32,907 |
| 49 | 60,508 | 124 | 1.83 | 3.15 |  | 75.36 | 32,881 |
| 50 | 59,779 | 126 | 1.83 | 3.03 |  | 74.22 | 32,824 |
| 51 | 58,833 | 143 | 1.83 | 2.91 |  | 73.02 | 32,803 |
| 52 | 57,781 | 140 | 1.83 | 2.77 |  | 71.26 | 32,567 |
| 53 | 56,856 | 162 | 1.83 | 2.63 |  | 69.03 | 32,275 |
| 54 | 56,069 | 173 | 1.83 | 2.51 |  | 66.75 | 31,850 |
| 55 | 55,499 | 192 | 1.83 | 2.39 |  | 64.16 | 31,518 |
| 56 | 54,772 | 200 | 1.83 | 2.29 |  | 60.89 | 30,930 |
| 57 | 54,614 | 212 | 1.83 | 2.20 |  | 57.44 | 30,268 |
| 58 | 54,953 | 202 | 1.83 | 2.13 |  | 53.12 | 29,448 |
| 59 | 55,931 | 253 | 1.82 | 2.07 |  | 48.09 | 28,405 |
| Total | 1,173,669 | 2,588 | 1.83 | 2.98 |  | 71.21 | 32,114 |

**Table A2** Sample sizes and frequencies or sample means for women who were diagnosed with breast cancer at ages 47-53.

| Age | Number of observations | Deceased | Number of adults | Household size |  | Employment | Individual income |
| --- | --- | --- | --- | --- | --- | --- | --- |
|  | Freq. | Freq. | Mean | Mean |  | % | Mean |
| 40 |  |  |  |  |  |  |  |
| 41 | 171 |  | 1.84 | 3.45 |  | 77.78 | 30,416 |
| 42 | 507 |  | 1.88 | 3.57 |  | 76.73 | 28,807 |
| 43 | 1,076 |  | 1.86 | 3.56 |  | 77.04 | 30,878 |
| 44 | 1,886 |  | 1.85 | 3.50 |  | 78.15 | 31,701 |
| 45 | 3,042 |  | 1.85 | 3.44 |  | 78.01 | 32,368 |
| 46 | 4,322 |  | 1.85 | 3.38 |  | 77.74 | 32,433 |
| 47 | 5,896 | 18 | 1.84 | 3.32 |  | 76.90 | 32,967 |
| 48 | 7,046 | 29 | 1.84 | 3.23 |  | 76.24 | 32,569 |
| 49 | 7,873 | 54 | 1.83 | 3.12 |  | 74.88 | 33,053 |
| 50 | 8,144 | 85 | 1.83 | 3.01 |  | 72.97 | 32,957 |
| 51 | 7,924 | 86 | 1.83 | 2.90 |  | 71.20 | 32,851 |
| 52 | 7,133 | 115 | 1.83 | 2.79 |  | 69.70 | 33,026 |
| 53 | 5,970 | 124 | 1.83 | 2.66 |  | 67.49 | 32,971 |
| 54 | 4,382 | 85 | 1.82 | 2.54 |  | 65.20 | 32,715 |
| 55 | 3,067 | 78 | 1.82 | 2.43 |  | 62.41 | 32,669 |
| 56 | 1,914 | 42 | 1.81 | 2.31 |  | 60.87 | 32,217 |
| 57 | 1,081 | 23 | 1.81 | 2.23 |  | 61.24 | 32,946 |
| 58 | 495 | NA | 1.79 | 2.15 |  | 58.59 | 32,552 |
| 59 | 148 | NA | 1.78 | 2.07 |  | 59.46 | 32,756 |
| Total | 72,077 | 751 | 1.84 | 2.98 |  | 72.04 | 32,719 |

Note: ‘NA’: the cell size is smaller than 10 and cannot be reported (data confidentiality).

**Table A3** Numbers for Fig. 1.

| Age of BC diagnosis (in full years) | Number of women |
| --- | --- |
| 47 | 1,211 |
| 48 | 1,202 |
| 49 | 1,355 |
| 50 | 2,040 |
| 51 | 1,818 |
| 52 | 1,466 |
| 53 | 1,423 |

**Table A4** Numbers for Figs. 2-4.

| Time around breast cancer (BC) diagnosis | BC-NP | NP-Counterfactual | BC-P | P-Counterfactual | DiD |
| --- | --- | --- | --- | --- | --- |
| years | ***Fig. 2: Mortality rate (%)*** | |  |  |  |
| 0 | 1.22 | 0.18 | 1.57 | 0.22 | 0.30 |
| 1 | 1.61 | 0.18 | 1.73 | 0.24 | 0.07 |
| 2 | 2.30 | 0.21 | 1.99 | 0.26 | -0.36 |
| 3 | 2.78 | 0.21 | 2.33 | 0.29 | -0.53 |
| 4 | 1.73 | 0.22 | 2.00 | 0.33 | 0.17 |
| 5 | 2.35 | 0.26 | 1.80 | 0.33 | -0.62 |
| 6 | 3.41 | 0.27 | 2.07 | 0.35 | -1.42 |
| years | ***Fig. 3: Employment rate (%)*** | |  |  |  |
| -6 | 77 | 76 | 78 | 75 | 1.75 |
| -5 | 76 | 77 | 77 | 75 | 1.86 |
| -4 | 77 | 77 | 77 | 75 | 2.04 |
| -3 | 78 | 77 | 76 | 75 | 0.50 |
| -2 | 78 | 77 | 76 | 74 | 0.84 |
| -1 | 78 | 77 | 75 | 74 | 0.59 |
| 0 | 75 | 76 | 71 | 72 | -0.39 |
| 1 | 72 | 76 | 68 | 71 | 0.71 |
| 2 | 70 | 75 | 66 | 70 | 1.17 |
| 3 | 69 | 75 | 63 | 69 | 0.71 |
| 4 | 68 | 74 | 63 | 67 | 1.69 |
| 5 | 66 | 73 | 62 | 65 | 3.06 |
| 6 | 66 | 71 | 59 | 63 | 1.57 |
| years | ***Fig. 4: Individual income (euro)*** | | |  |  |
| -6 | 30,240 | 30,516 | 31,332 | 30,660 | 948 |
| -5 | 30,378 | 30,918 | 32,063 | 31,246 | 1,356 |
| -4 | 31,119 | 31,447 | 31,907 | 31,705 | 530 |
| -3 | 32,138 | 31,902 | 32,868 | 32,146 | 487 |
| -2 | 33,185 | 32,403 | 33,209 | 32,482 | -55 |
| -1 | 33,052 | 32,698 | 33,544 | 32,672 | 518 |
| 0 | 32,824 | 32,900 | 33,407 | 32,717 | 767 |
| 1 | 31,795 | 33,199 | 32,638 | 32,758 | 1,284 |
| 2 | 32,545 | 33,423 | 32,971 | 32,716 | 1,133 |
| 3 | 32,723 | 33,532 | 33,167 | 32,644 | 1,333 |
| 4 | 33,337 | 33,459 | 32,829 | 32,356 | 596 |
| 5 | 31,905 | 33,215 | 32,407 | 32,115 | 1,602 |
| 6 | 32,978 | 33,023 | 31,511 | 31,575 | -18 |

Notes: The four groups of women are: BC-P (BC-NP) for women diagnosed with breast cancer and who were (not) covered by the program, and P-Counterfactual (NP-Counterfactual) for women who were not diagnosed with breast cancer and in the same years were of the same ages as women in the BC-P (BC-NP) group. DiD = BC-P - P-Counterfactual - (BC-NP - NP-Counterfactual), which is a crude point estimate of the program effect based on the aggregate numbers.
